# Phase Identification in a Scanning Electron Microscope Using Backscattered Electron Kikuchi Patterns

**DOI:** 10.6028/jres.101.031

**Published:** 1996

**Authors:** R. P. Goehner, J. R. Michael

**Affiliations:** Materials and Process Sciences Center, Sandia National Laboratories, Albuquerque, NM 87185-1405

**Keywords:** electron diffraction, Kikuchi patterns, scanning electron microscopy

## Abstract

Backscattered electron Kikuchi patterns (BEKP) suitable for crystallographic phase analysis can be collected in the scanning electron microscope (SEM) with a newly developed charge coupled device (CCD) based detector. Crystallographic phase identification using BEKP in the SEM is unique in that it permits high magnification images and BEKPs to be collected from a bulk specimen. The combination of scanning electron microscope (SEM) imaging, BEKP, and energy dispersive x-ray spectrometry holds the promise of a powerful new tool for materials science.

## 1. Introduction

The identification of unknown micron-sized phases in the SEM has been limited by the lack of a robust and simple way to obtain crystallographic information about the unknown while observing the microstructure of the specimen. A variety of techniques are available that can provide some information about the identity of unknown phases. For example, energy dispersive x-ray spectrometry (EDS) is of some use but obviously cannot distinguish between phases of similar compositions but different crystal structures (an example of this is TiO_2_ that has two tetragonal forms with different atomic arrangements and an orthorhombic form). Other techniques can provide the required information but have significant limitations. Micro area x-ray diffraction techniques are capable of identifying crystalline phases, but cannot be used to identify sub-micron sized areas. Selected area electron diffraction in the transmission electron microscope (TEM) can provide crystallographic information from micron-sized regions of the specimen, but TEM requires electron-transparent thin specimens to be produced, which is time consuming and can be very difficult. In this paper we will describe a new charge coupled device (CCD) based camera and then demonstrate that BEKP in the SEM using this camera can provide crystallographic phase identification of sub-micron sized areas with little or no difficult specimen preparation.

The first backscattered electron Kikuchi patterns were observed nearly 40 years ago before the development of the SEM [1]. The patterns were recorded using a special chamber and photographic film. The addition of an appropriate camera system to an SEM for the observation of BEKPs in a modern SEM was demonstrated in the 1970s [2]. Since the late 1970s, BEKPs have been used for the accurate determination of crystallographic orientation of sub-micron crystals [3]. The determination of crystallographic texture from BEKPs does not require high quality patterns and can be performed on-line with a conventional video camera system [4].

BEKP has been used to identify crystal symmetry elements and crystallographic point groups. This work demonstrated that 27 of the 32 possible point groups could be distinguished using BEKP [5–9]. The pattern quality required for studies of this type dictated that the patterns be recorded on photographic film with all of the inherent difficulties of film use in a vacuum chamber. Recent work has shown that on-line phase identification can be achieved through the use of BEKP with a CCD camera [11, 12].

The major advantage of BEKP over conventional powder diffraction is the ability in the SEM to image a feature of interest in either secondary electron or backscattered electron imaging modes and then obtain crystallographic phase information and compositional information using EDS from this feature. BEKP can provide phase information on specimens in reflection with little or no sample preparation in very short periods of time (i.e., on the order of minutes) on features below the resolution limits of microdiffraction. A considerable difficulty with microdiffraction is the problem of accurately viewing the area of interest and assuring that the x-ray beam is positioned on the area of interest. Microdiffraction uses two separate sets of optics, a traveling microscope for viewing the sample and positioning the x-ray beam and a collimator that delivers x rays to the specimen. These two sets of optics view the specimen from separate directions and at different angles and thus require very accurate alignment in order to ensure that the light optics and the x-ray collimator are coincident upon the specimen. BEKP uses one set of optics for both viewing and analyzing the specimen. Another difficulty with microdiffraction is the requirement that the material be polycrystalline or that the sample be oscillated and rotated in order to yield a pseudo-polycrystalline x-ray pattern. As the specimen is rotated and oscillated the x-ray beam can move off of the feature of interest. BEKP does not require specimen motion to collect a pattern. A detailed description of the technique and comparisons to electron channeling patterns, transmission electron diffraction patterns and micro x-ray diffraction techniques is described elsewhere [11]. A disadvantage of BEKP is its inability to distinguish between very fine grained (< 0.1 μm) or amorphous material.

## 2. Theory

Backscattered electron Kikuchi patterns are obtained by illuminating a highly tilted specimen with a stationary electron beam. The beam electrons are elastically and inelastically scattered within the specimen with some of the electrons scattered out of the specimen. These backscattered electrons form the diffraction pattern. There are currently two mechanisms used to describe the formation of these patterns. In the first description, Kikuchi patterns are formed by the elastic scattering (diffraction) of previously inelastically scattered electrons [1]. These backscattered electrons appear to originate from a virtual point below the surface of the specimen. Some of the backscattered electrons which satisfy the Bragg condition (+ *θ* and −*θ*) are diffracted into cones having a semi-angle of (90°–*θ*), with the cone axis normal to the diffracting plane [4]. Since the wavelength for 20 kV–40 kV electrons is small, the Bragg angle (*θ*) is less than 2°. These pairs of very flat cones intersect the detector and are imaged as two nearly straight Kikuchi lines separated by 2*θ* [13]. An alternative description of the pattern formation requires one high-angle scattering event that results in an electron exiting the specimen. The channeling of these electrons by the crystallographic planes of the specimen results in the formation of cones of intensity in a manner analogous to the channeling of electrons in electron channeling patterns [14]. Fortunately, we do not require a detailed understanding of the physics of electron scattering to use these patterns for phase identification in the SEM. The intensities of the Kikuchi lines are proportional to the structure factor to second power (*F*^2^) and do not vary significantly as the crystal orientation is changed. This insensitivity of intensity to orientation is an important property that make BEKPs easily applicable to crystallographic phase analysis.

## 3. Experimental

In order to overcome the disadvantages of photographic film and video rate recording of the patterns, a camera was developed based on CCD technology. This camera consists of a thin yttrium aluminum garnet (YAG) scintillator that is fiber-optically coupled to a scientific-grade thermoelectrically-cooled slow-scan CCD. A CCD with 1024 × 1024 pixels was chosen for this application to achieve high accuracy in the measurement of the recorded BEKP. In order to achieve a large collection angle the CCD, 2.54 cm × 2.54 cm, was coupled to the scintillator through a 2.5:1 fiber-optic reducing bundle. The front surface of the YAG scintillation was parallel to the electron beam and between 40 mm to 60 mm from the specimen resulting in a maximum collection angle of over 70° [11, 12].

The CCD is a good replacement for photographic film recording of BEKPs. Typically, high quality CCDs have a higher dynamic range than photographic film, but the resolution of the CCD still cannot approach the resolution of photographic film [19, 20]. However, for images collected at a resolution of 1024 × 1024, it is difficult for the unaided eye to resolve the individual pixels. For phase identification using BEKP in the SEM, CCD detectors are superior to film due to the high dynamic range of the CCD and their adequate resolution and the immediate availability of the image for analysis [15].

BEKP have been obtained from a range of materials. A tilted specimen is imaged using either the backscattered or the secondary electron signal to select a feature of interest and the electron beam is stopped on the area, just as one would use a stationary beam to obtain EDS information. The exposure time is controlled by un-blanking the electron beam for the required time. The CCD detector configuration permits high quality patterns to be recorded using exposures of 1 s to 10 s [11]. The actual exposure depends on the specimen (atomic number) and the electron optical conditions (accelerating voltage and beam current). Patterns have been successfully recorded at accelerating voltages as low as 2.5 kV.

**Fig. 1:**
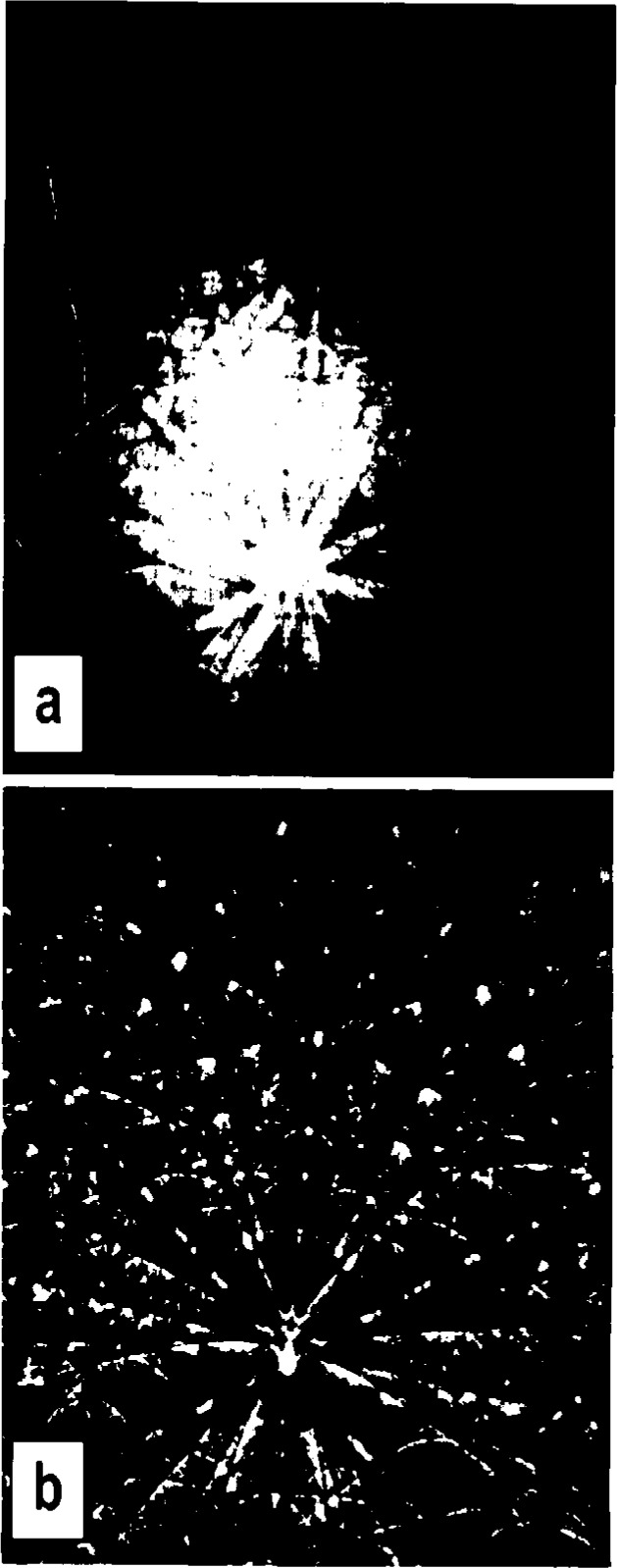
Flat fielding procedure can greatly increase the contrast and quality of BEKP images, a) raw image of Mo at 40 kV before flat fielding, b) image after flat fielding.

The patterns are improved significantly by an image-processing procedure called flat fielding [16]. The raw patterns collected with the CCD consist of relatively weak but detailed crystallographic information superimposed on a slowly changing background intensity and image artifacts related to the fiber-optic bundle and the scintillator. Once a raw image has been recorded a second image (the flat-field image) is recorded that contains the image artifacts but not the crystallographic information. This image may be obtained by scanning the beam over a number of crystals while collecting a pattern or by recording patterns from very fine grained materials. The image artifacts and the slowly varying background component can be removed by normalizing the raw image containing the crystallographic information with the flat field image. This procedure has the added advantage of increased contrast in the processed patterns. An example of the flat-fielding procedure is shown in Fig. 1 a and b.

**Fig. 2:**
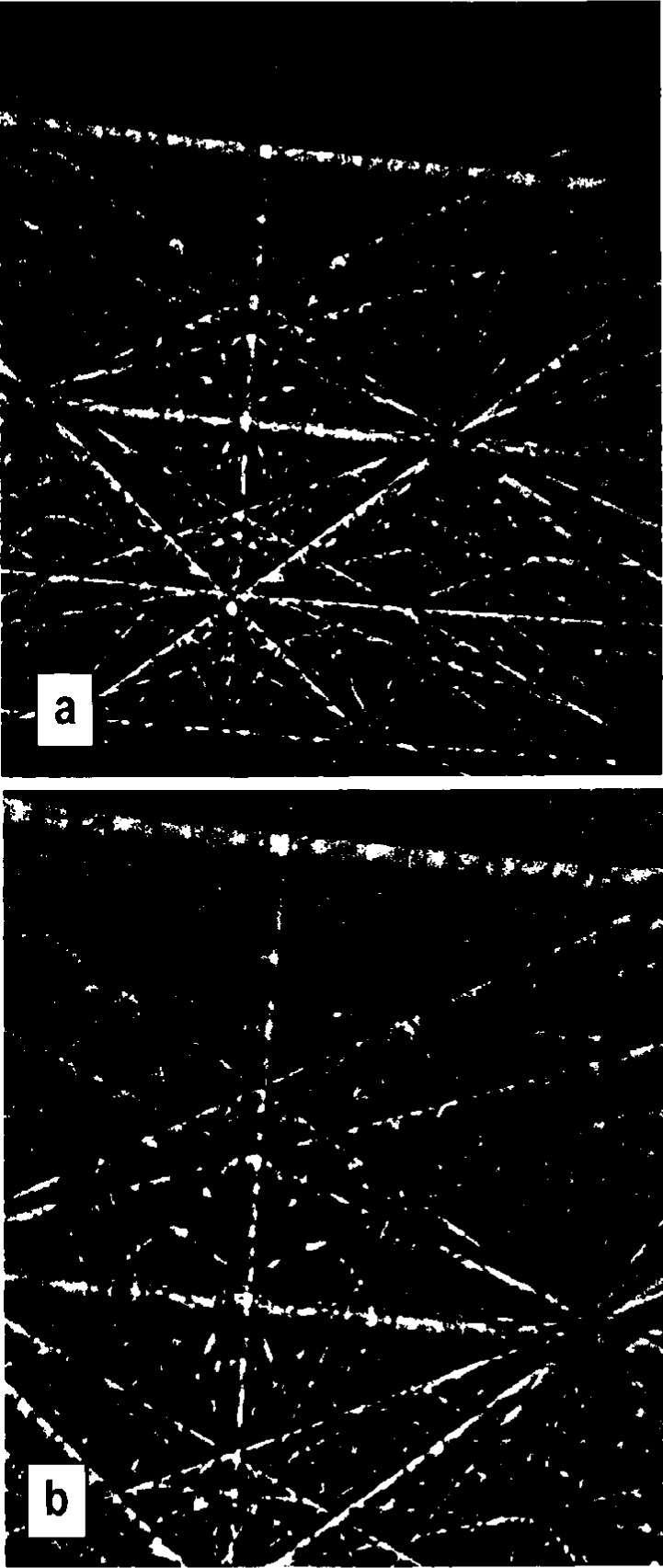
BEKPs obtained from Si at 40 kV for two different camera lengths, a) long camera length, b) short camera length.

**Fig. 3:**
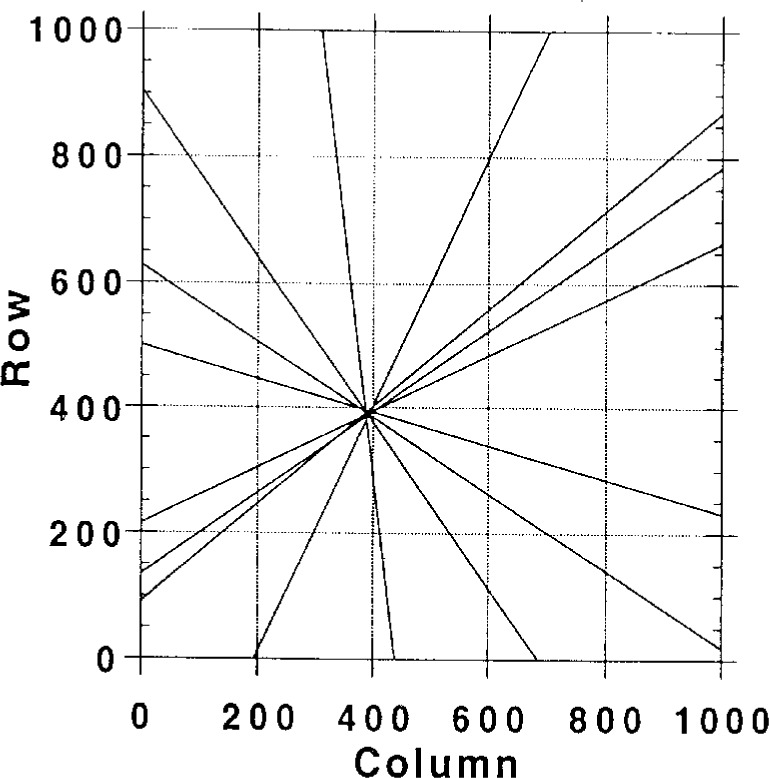
Plot of the lines connecting the same poles in the short and long camera length images of Si shown in Fig. 2. The lines intersect at a common point that represents the pattern center.

The accurate determination of crystallographic parameters from BEKPs requires that the camera system be carefully calibrated [16]. The two parameters that require calibration are the pattern center and the specimen-to-detector distance. The pattern center is defined as the projection of the beam impact point onto the scintillator. There are a number of ways to calibrate the pattern center. We have found that one of the most accurate and simple ways to accomplish this is to record patterns from the same crystal at two different scintillator-to-specimen distances [11]. Si patterns taken at long and short detector-to-specimen distances are shown in Fig. 2 a and b. The crystallographic zones move along radial lines that project out from the pattern center. It is quite simple to plot the positions of the zones as they move to determine the pattern center. Figure 3 shows a plot of the radial lines extracted from the patterns in Fig. 2 a and b. The pattern center is located where these lines intersect. This technique has been used to determine the pattern center to within one or two pixels. The CCD camera has been calibrated using this procedure and has been found to be extremely stable with time. Once the pattern center is accurately known the specimen-to-detector distance can be easily determined from a known specimen. Table 1 shows the measured angles between zones for Si recorded at 30 kV and compares the measured values with values calculated from the crystal structure. There are several other methods have been used for calibration, but the procedure described appears to be the most reliable [17–19].

## 4. Results and Discussion

Phase identification with BEKP in the SEM has previously been accomplished by recording patterns on film. On-line phase identification has not been possible due to the necessity to expose and develop the photographic film. The CCD detector system eliminates this problem, as the patterns are immediately available for viewing and analysis and are of similar quality to those obtained on photographic film. Phase identification is achieved in the following manner.

First an area of interest is located on the tilted specimen using the appropriate imaging signal (backscattered or secondary electrons). A BEKP is then collected from the feature of interest using a stationary beam in the same way that x-ray information is obtained. A flat field image is then obtained and used to flat field the raw image data as has been described previously. Compositional data is also obtained from the feature of interest as this information is used to limit the number of possible candidate materials that could produce the BEKP.

The next step in phase identification using BEKP is to determine the angles between the various zone axes present in the image. Candidate crystal structures, consistent with the compositional information obtained from the unknown, are selected from the literature. The measured interzonal angles are then compared with those calculated from the candidate crystal structure. Quite often these angles and the compositional information are sufficient to unequivocally identify the unknown crystalline phase. In order to simplify this procedure a number of tools have been developed. A program was developed that determines the indices of the directions of designated zones observed in the BEKP. The program utilizes a file of significant zones which is generated by calculating zones that are at the intersection of planes having significant intensity usually defined as a relative intensity of 20–100. The analysis provides the *x* and *y* location of four significant zones, the lattice constants of the crystal, and the file of significant zones. The program then resolves the observed angle between the zones with the calculated angles between the file of significant zones. The program reports all sets of consistent zones that fit within a designated error (usually 2°) for the four entered zone locations.

The significant zones are determined from the cross product of planes of significant intensity, thus the intersection of at least two planes of significant intensity would result in a zone that would be a candidate for the indexing program. Files of significant zones have been developed for many common materials including generic files for diamond, face centered cubic, body centered cubic, and hexagonal close packed structures. A database could be developed for structure types containing significant zones. This database could then form the bases of an automated phase identification program.

Once a possible match is found, the pattern is simulated in the correct orientation. A program was developed that simulates the patterns and includes the geometric distortions that result from the gnomonic projection. The software simulates the patterns by generating the reciprocal lattice for a triclinic cell as has been done previously for single-crystal Laue and selected area electron-diffraction patterns [20, 21]. The triclinic crystal system is used for the simulation because it is the general case and all crystal systems are special cases of the triclinic cell. Reciprocal points are generated for the lattice constants (*a*, *b*, *c*, *α*, *β*, ∂) allowed by the centering conditions such as base centered, body centered, and face centered. Cones with a semi angle of (90–*θ*) are constructed about each reciprocal lattice point. The intersection of the diffracted cones and the planar detector result in the conic sections observed in the BEKP. The intensity of the Kikuchi lines has been observed to be similar to the intensities calculated for electron diffraction patterns using the diffraction intensity calculating program POWDER [22]. These calculated intensities are used by the simulation program to control the width of the Kikuchi lines in the simulated patterns. The simulated pattern is compared to the experimentally obtained pattern and if the agreement between the experimental and the simulated pattern is good, then an identification is assumed to have been accomplished.

It should be noted that in a vast majority of cases there is no need to make use of the interplanar spacings that can be measured from the BEKPs. Cubic materials present the most difficulty in using the approach described above because the interzonal angles do not vary with the lattice spacing of the crystal. As the symmetry of the crystal decreases the angles between the zone axes in the pattern becomes more diagnostic. Fortunately, it is possible to measure the interplanar spacings from a BEKP. This is done by measuring the distance between the pair of lines that produce the Kikuchi band and converting this angle to a interplanar spacing [4]. This data can then be used as a further condition for identifying the unknown phase of interest and is particularly useful for cubic crystal structures.

## 5. Examples of Phase Identification Using BEKP

### 5.1 Identification of M_23_C_6_ and M_6_C an Austenitic Alloy

**Fig. 4:**
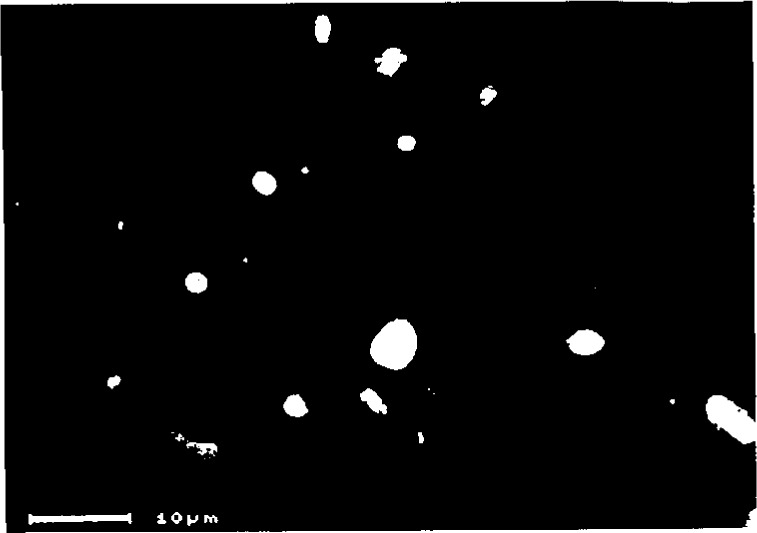
Backscattered electron image of a metallographically prepared creep tested Ni-based austenitic alloy that shows the presence of two precipitate phases. The brighter imaging phase was shown by EDS to be W-rich and the medium gray phase was shown to be Cr-rich.

**Fig. 5:**
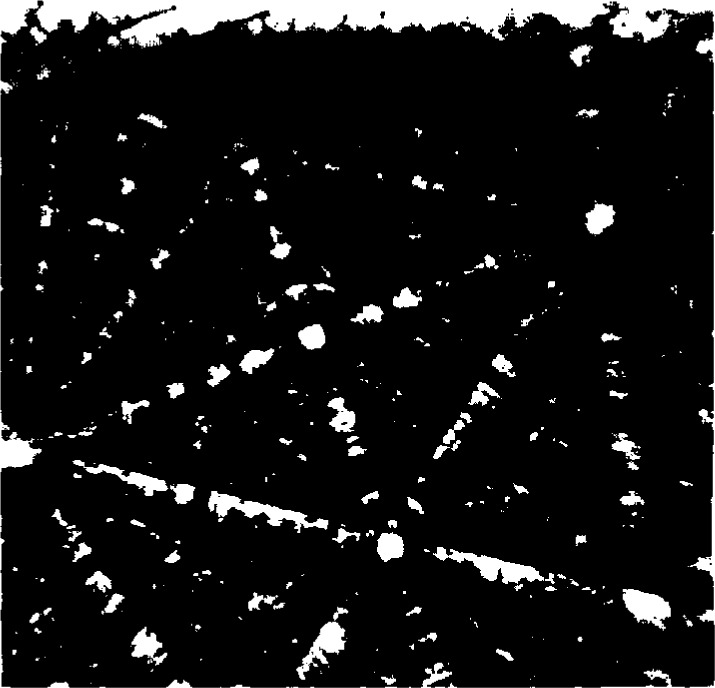
BEKP of the white phase (W rich) obtained at 30 kv.

**Fig. 6:**
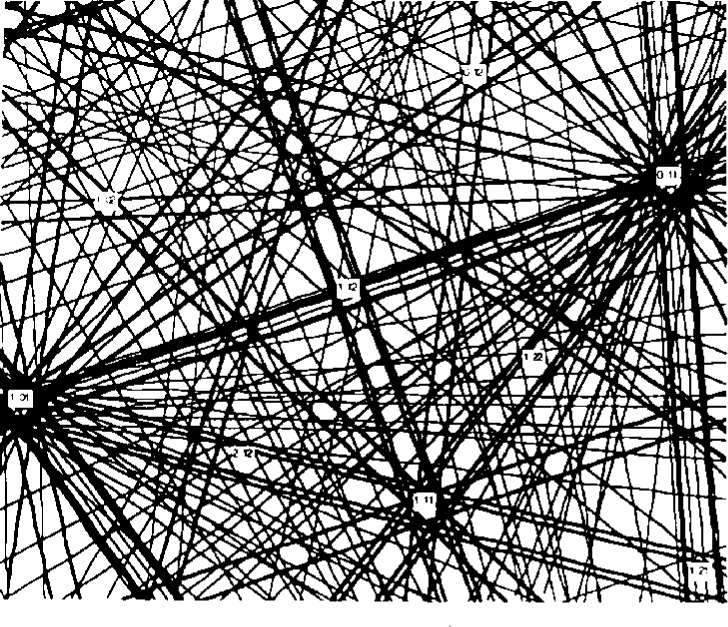
Simulation of M_6_C, using Co_3_W_3_C as a structure model, and shows an exact match with the experimental BEKP shown in Fig. 5.

**Fig. 7:**
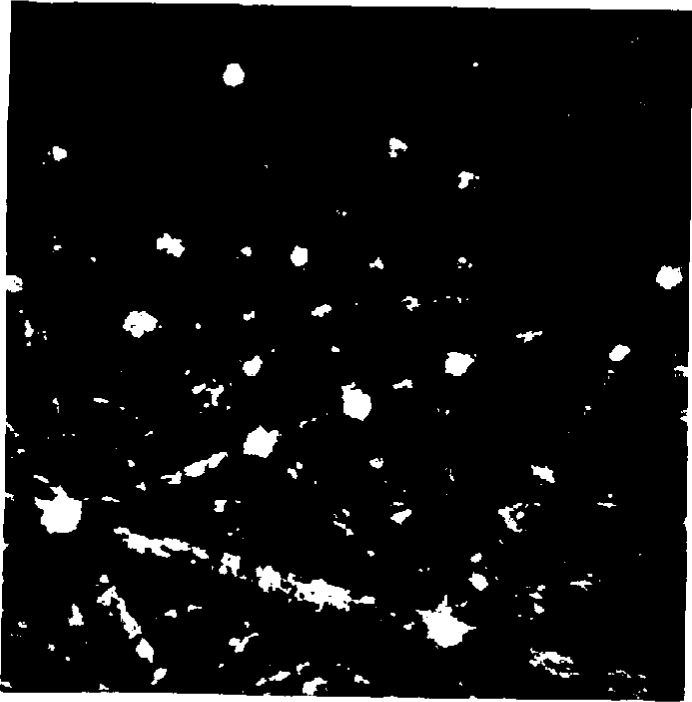
BEKP of the gray phase (Cr rich) obtained at 30 kV.

**Fig. 8:**
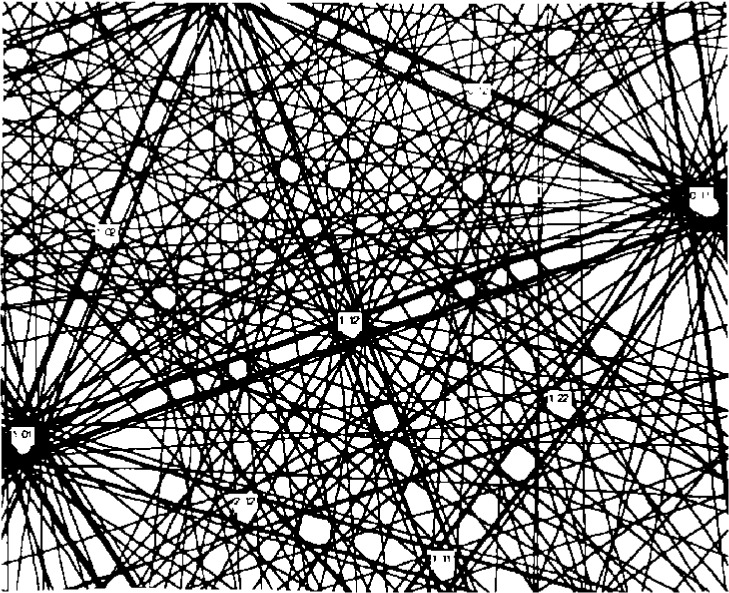
Simulation of M_23_C_6_, using Cr_23_C_6_ as a structure model, and shows an exact match with the experimental BEKP shown in Fig. 7.

Figure 4 is a backscattered electron image of a metallographically prepared creep-tested Ni-based austenitic alloy that shows the presence of two precipitate phases. The brighter imaging phase was shown by EDS to be W-rich and the medium gray phase was shown to be Cr-rich. These precipitates were suspected to be metal carbides. Figure 5 is the BEKP of the white phase which indexed consistently as an M_6_C phase (where M designates a transition metal). Co_3_W_3_C (PDF 27-1125) was used as the structure model [23]. The simulation of M_6_C in the proper orientation, as determined by the indexing software, is shown in Fig. 6 and shows an exact match with the experimental BEKP. Figure 7 is the BEKP of the gray phase which was indexed as M_23_C_6_. Cr_23_C_6_ (PDF 35-783) was used as the structure model [23]. Figure 8 shows the exact match between the simulated and experimental image. This example shows that BEKPs can be used to distinguish between phases with similar structures. It is interesting to note that both of these crystal structures are cubic and that M_23_C_6_ is face centered cubic while M_6_C is diamond cubic. M_23_C_6_ belongs to the space group Fm3m (225) with a lattice constant of 10.66 Å and can be easily distinguished from M_6_C that belongs to the space group Fd3m (227) with a lattice constant of 11.11 Å [23].

### 5.2 Identification of Laves Phase in Weld Microstructure

**Fig. 9:**
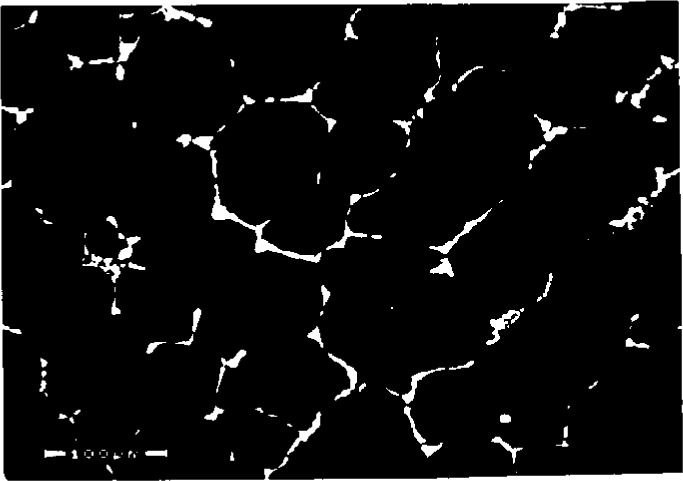
Backscattered electron image of the simulated weld. EDS x-ray analysis in the SEM demonstrated that the second phase was rich in Fe and Nb.

During the simulated welding of an Fe-Co-Ni-Cr-Nb alloy a second phase was observed in metallographically prepared sections. Figure 9 is a backscattered electron image of the simulated weld that shows the precipitates that image brighter than the matrix phase. EDS x-ray analysis in the SEM demonstrated that the second phase was rich in Fe and Nb.

**Fig. 10:**
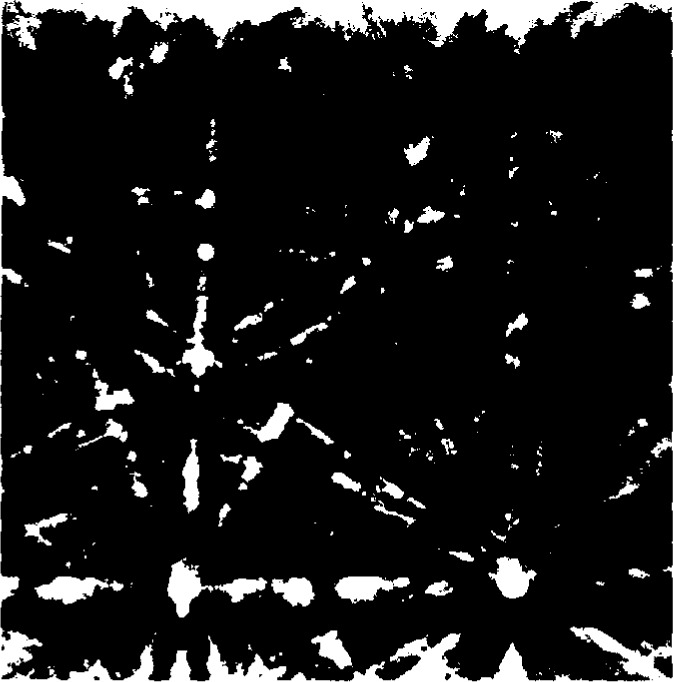
BEKP collected at 30 kV in the SEM from the bright precipitates in the simulated weld shown in Fig. 9.

**Fig. 11:**
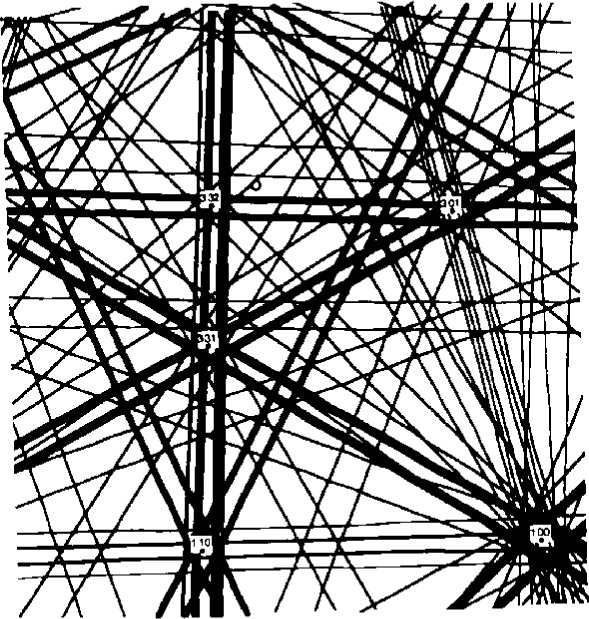
Simulated BEKP for Fe_2_Nb that demonstrates good agreement with Fig. 10.

BEKPs of the bright imaging phase were obtained from a metallographically polished, but not etched sample. The SEM was operated at 30 kV with a beam current of 1 nA–2 nA. Figure 10 shows an example of the BEKP from the bright precipitates in the simulated weld. A survey of the literature showed that there were a number of phases that contain Fe and Nb. Possible Nb-Fe phases were NbFe, Fe_2_Nb, and Fe_7_Nb_6_ [23]. Interzonal angles observed in the experimental patterns were measured and compared with the calculated values for the above three compounds. Good agreement was found between the interzonal angles for Fe_2_Nb and the experimentally measured interzonal angles. Patterns for Fe_2_Nb were simulated, including the effects of structure factors, and the simulation was compared with the experimental patterns. Figure 11 is a BEKP simulation for Fe_2_Nb calculated using the same microscope parameters as those used for collecting the BEKP from the unknown bright phase. As can be seen from comparison of Fig. 10 and Fig. 11 the agreement is excellent. Thus, the bright phase was identified as Fe_2_Nb, a Laves phase that is hexagonal (*a* = 0.4837 nm and *c* = 0.7884 nm).

## 6. Conclusion

The combination of BEKP and EDS now permits unequivocal identification of micron sized areas in the SEM. The patterns contain a wealth of information about the specimen and some that as yet, has not been taken advantage of. It should be possible to utilize one or more BEKPs to calculate a reduced cell that could be used to search the crystal data in a more automated manner than is presently used. Although unequivocal phase identification is not possible in the most general sense, the combination of diffraction information from BEKP and compositional information from EDS presents a powerful tool for materials analysis.

## Figures and Tables

**Table 1:** Comparison of measured interzonal angles with actual angles for Si

Angle between	Measured (°)	Actual (°)
[001]–[111]	54.81	54.7
[001]–[011]	44.93	45.0
[011]–[112]	29.94	30.0
